# Fibromodulin overexpression drives oral squamous cell carcinoma via activating downstream EGFR signaling

**DOI:** 10.1016/j.isci.2023.108201

**Published:** 2023-10-13

**Authors:** Lingyun Xia, Tianshu Zhang, Juncheng Yao, Kaitian Lu, Ziqiu Hu, Xinsheng Gu, Yongji Chen, Shanshan Qin, Weidong Leng

**Affiliations:** 1 Department of Stomatology, Taihe Hospital, Hubei University of Medicine, Shiyan 442000, P.R. China; 2 Institute of Oral Diseases, School of Dentistry, Hubei University of Medicine, Shiyan 442000, P.R. China; 3 Hubei Key Laboratory of Embryonic Stem Cell Research, School of Basic Medical Sciences, Hubei University of Medicine, Shiyan 442000, Hubei, China

**Keywords:** Cell biology, Cancer

## Abstract

Accumulating evidence has shown that fibromodulin (FMOD) plays a pivotal role in tumorigenesis and metastasis. However, the biological function of FMOD in oral squamous cell carcinoma (OSCC) remains largely unclear to date. In this study, we confirmed that FMOD was overexpressed and showed a significant association with malignant progression and lymph node metastasis in OSCC. Depletion of FMOD inhibited OSCC proliferation and metastasis *in vitro* and *in vivo*. RNA sequencing, western blotting, and rescue assays verified that FMOD exerted oncogenic roles in OSCC via activation of EGFR signaling. In addition, FMOD was proved to be a putative target gene of miR-338-3p. Taken together, FMOD overexpression due to the reduced level of miR-338-3p promotes OSCC by activating EGFR signaling. Our findings provide direct evidence that targeting FMOD could be a promising therapeutic strategy for OSCC patients.

## Highlights

• FMOD mRNA and protein are upregulated in OSCC
• FMOD overexpression is closely correlated with OSCC progression and metastasis
• FMOD depletion inhibits OSCC by blocking EGFR signaling
• FMOD mRNA is directly targeted by miR-338-3p

## Graphical abstract

**Figure:**
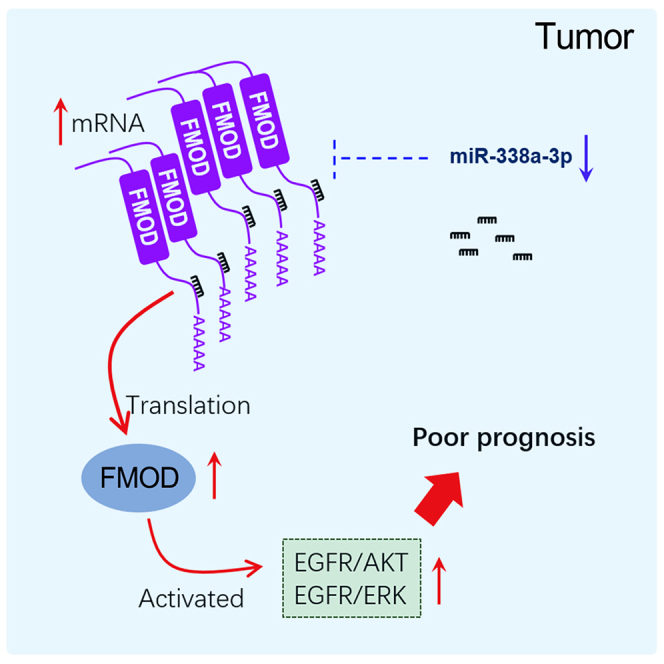

## Introduction

Oral squamous cell carcinoma (OSCC) constitutes approximately 90% of head and neck squamous cell carcinoma (HNSCC) and is the sixth most common cancer in the world.^1^ The prognosis of OSCC patients remains unsatisfactory since the 5-year survival rate is only 50–60%.^2^ Due to the low rate of early diagnosis, patients are diagnosed at an advanced stage with metastasis, and treatment strategies are limited.^3^^,^^4^^,^^5^ Therefore, the identification of biomarkers responsible for early diagnosis of OSCC is helpful to obtain better clinical outcomes for OSCC patients.^6^

Fibromodulin (FMOD), belonging to Class II small-leucine-rich proteoglycan (SLRPs), is an extracellular matrix (ECM) protein.^7^ As a secreted protein, FMOD was originally found to be involved in the regulation of collagen fiber formation.^8^ FMOD participates in the reconstruction of ECM through combining with matrix molecules and plays an essential role in connective tissues such as skin, cartilage, tendon, sclera, and cornea.^9^^,^^10^ Recent studies have shown that FMOD plays an vital role in the occurrence and development of malignant tumors.^11^ For example, Ao Zhi et al.^12^ found that FMOD was highly specifically expressed in small cell lung cancer (SCLC), and the expression level was positively correlated with tumor angiogenesis in SCLC. Another study on non-small cell lung cancer H322 cells showed that the silencing of FMOD expression might significantly inhibit the proliferation, adhesion, and migration of H322 cells by inhibiting the TGF-β/Smad signaling pathway.^13^ FMOD knockdown could significantly inhibit Erk phosphorylation expression, cell migration, and invasion in breast cancer cells.^14^ Reyes et al.^15^ found that FMOD was highly expressed in prostate cancer tissues. However, the expression pattern and biological role of FMOD remains largely unclear in OSCC.

Increasing evidence has indicated that FMOD was involved in regulating certain signaling pathways. For example, FMOD plays a role in regulating TGF-β signaling and NFkB pathways.^16^ Zheng et al. have reported that FMOD critically coordinates the temporospatial distribution of TGF-β ligands and receptors and modulates TGF-β bioactivity in a complex way beyond simple physical binding to promote proper wound healing.^17^ In addition, Lee et al. have shown that FMOD suppresses NFkB activity by stabilizing IKBA.^18^ In this study, we unravel a critical role of FMOD in regulating cellular EGFR signaling. Our finding highlighted that miR-338-3p downregulation led to FMOD overexpression, thereby driving OSCC progression through activating EGFR signaling.

## Results

### FMOD mRNA and protein were overexpressed in OSCC tissues and cell lines

**Figure 1:**
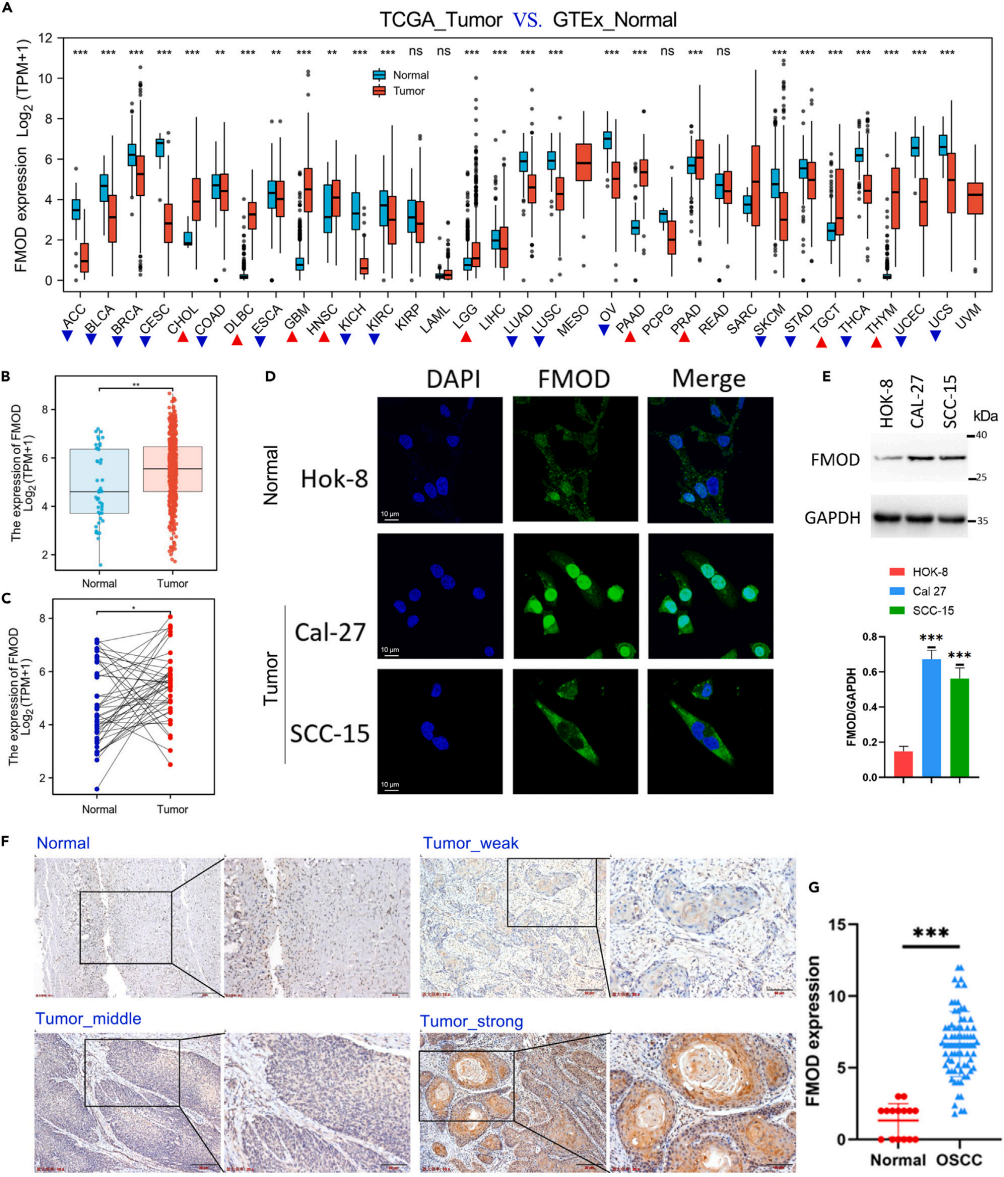
FMOD was significantly overexpressed in OSCC (A) Pan-cancer analysis of FMOD expression using TCGA database. The red triangle represents upregulation, while the blue triangle represents downregulation. (B) The FMOD mRNA level (FPKM value) in GTEx normal tissues and TCGA_OSCC tumor tissues. (C) The FMOD mRNA level (FPKM value) in 43 paired OSCC tissues in the TCGA cohort. (D) Immunofluorescence analysis revealed the subcellular location of FMOD protein in OSCC cell lines (SCC-15 and CAL-27) and normal oral cells (HOK-8). (E) WB analysis of FMOD protein in OSCC cell lines (SCC-15 and CAL-27) and normal oral cells (HOK-8). The bar graph shows that FMOD was expressed in CAL-27 and SCC-15 cells. (F) Representative immunohistochemical staining of FMOD in oral normal tissue and OSCC. FMOD was undetectable in 7 samples and very weakly expressed in 8 samples. (a) FMOD staining in normal oral tissues. (b) Weak FMOD staining in #16 OSCC, TNM: I, T1N0M0. (c) Medium FMOD staining in #43 OSCC, TNM: IV, T3N2M0. (d) Strong FMOD staining in #31 OSCC, TNM: IV, T2N2M0. (G) Expression levels of FMOD were higher in OSCC than those of the normal oral tissues. ∗, p < 0.05; ∗∗, p < 0.01; ∗∗∗p < 0.001.

To investigate the mRNA level of *FMOD* gene in non-cancerous tissues and cancerous tissues, pan-cancer analysis was conducted using TCGA datasets. We used GTEx_normal tissues as a control. The results showed that FMOD mRNA was abnormally expressed in most cancers (Figure 1A). Notably, FMOD transcripts were significantly upregulated in HNSCC (Figure 1A). To further examine FMOD mRNA level in OSCC, we performed expression analysis in TCGA_OSCC cohort. Consistently, FMOD mRNA level was significantly upregulated in OSCC (Figures 1B and 1C).

On the other hand, we conducted immunofluorescence and immunoblotting assays in normal oral epithelial keratinocytes (HOK-8) and OSCC cell lines (SCC-15, CAL-27) to detect the expression and subcellular localization of FMOD. The results showed that FMOD protein was distributed in both the nucleus and cytoplasm and was significantly upregulated in OSCC cell lines (Figures 1D and 1E). Additionally, the immunohistochemistry (IHC) assay in 10 normal oral tissues and 77 OSCC tissues was performed to determine the expression of FMOD. The results showed that FMOD expression was scored 0 (7 samples) or weak (8 samples) in the cell membrane, nuclei, and cytoplasm of the cells in 15 normal non-cancerous oral sample tissues (Figure 1F). The levels of FMOD protein in OSCC tissues varied. A total of 54 tissue samples exhibited strong expression, 20 tissue samples exhibited medium expression, and 3 tissue samples exhibited weak expression of FMOD (Figure 1F). Statistical results showed that FMOD protein was significantly overexpressed in OSCC (Figure 1G, p < 0.001).

### FMOD overexpression is clinically associated with tumor stage and metastasis in OSCC

To understand the clinical significance of FMOD overexpression, we performed a correlation analysis between FMOD expression and clinical characteristics of 77 OSCC tissues using the Pearson Chi-square test. The results showed no significant correlation between FMOD expression and gender, age, smoking history, drinking history, or pathological grade (Table 1).

**Table 1:** Clinicopathological significance of FMOD expression in OSCC

Characteristic	FMOD expression				
	Case (n=77)(%)	Weak (n=3)(%)	Medium (n=20)(%)	Strong (n=54)(%)	P value
**Gender**					

Male	48(62.34)	1(33.33)	11 (55.00)	36(66.67)	0.374
Female	29(37.66)	2(66.67)	9 (45.00)	18(33.33))	

**Age at diagnosis (years)**					

≥60	43(55.84)	1(33.33)	10(50.00)	32(59.26)	0.563
＜60	34(44.16)	2(66.67)	10(50.00)	22(40.74)	

**Smoking**^a^					

yes	44(57.14)	2(66.67)	12(60.00)	30(55.56)	0.890
no	33(42.86)	1(33.33)	8 (40.00)	24(44.44)	

**Alcohol**^a^					

yes	51(66.23)	2(66.67)	13(65.00)	36(67.67)	0.991
no	26(33.77)	1(33.33)	7 (35.00)	18(33.33)	

**Grade**					

G1	46(59.74)	2(66.67)	12(60.00)	32(59.26)	0.825
G2	27(35.06)	1(33.33)	6 (30.00)	20(37.04)	
G3	4 (5.20)	0(00.00)	2 (10.00)	2 (3.70)	0.968
G1	46(59.74)	2(66.67)	12(60.00)	32(59.26)	
G2+G3	31 (40.26)	1(33.33)	8 (40.00)	22(40.74)	

**Clinical stage (TNM)**					

I	15(19.48)	1(33.33)	5 (25.00)	9 (16.67)	0.100
II	20(25.97)	2(66.67)	8 (40.00)	10(18.52)	
III	10(12.99)	0(0.00)	2 (10.00)	8 (14.81)	
IV	32(41.56)	0(0.00)	5 (25.00)	27(50.00)	0.011
I+II	35(45.45)	3(100.00)	13(65.00)	19(35.19)	
III+IV	42(54.55)	0(0.00)	7 (35.00)	35(64.81))	

**T stage**					

T1	16(20.78)	1(33.33)	5 (25.00)	10(18.52)	0.692
T2	33(42.86)	2(66.67)	9 (45.00)	22(40.74)	
T3	16(20.78)	0(0.00)	4 (20.00)	12(22.22)	
T4	12(15.58)	0(0.00)	2 (10.00)	10(18.52)	0.285
T1+T2	49(63.64)	3(100.00)	14(70.00)	32(59.26)	
T3+T4	28(36.36)	0(0.00)	6 (30.00)	22(40.74)	

**N stage**					

N0	47(61.04)	3(100.00)	16(80.00)	28(51.85)	0.086
N1	7 (9.09)	0(0.00)	1 (5.00)	6 (11.11)	
N2	23(29.87)	0(0.00)	3 (15.00)	20(37.04))	0.032
N0	47(61.04)	3(100.00)	16(80.00)	28(51.85)	
N1+N2	30(38.96)	0(0.00)	4 (20.00)	26(48.15)	

a The study subjects with a smoking and drinking habits were classified as smokers and drinkers (at least once a week). The Chi-square test was used to analyze each group of data to obtain P values.

Interestingly, OSCC patients with high expression of FMOD tend to be associated with middle clinical stages (III+IV), while patients in the early (I + II) stage possessed low expression of FMOD (p = 0.011, Table 1). Further analysis showed that FMOD expression was significantly correlated with lymph node metastasis (N0 and N1+N2) in OSCC patients (p = 0.032, Table 1). These results suggested that aberrant FMOD overexpression was an indicator of malignant progression and lymph node metastasis in OSCC.

### Knockdown of FMOD significantly hindered OSCC cell proliferation

**Figure 2:**
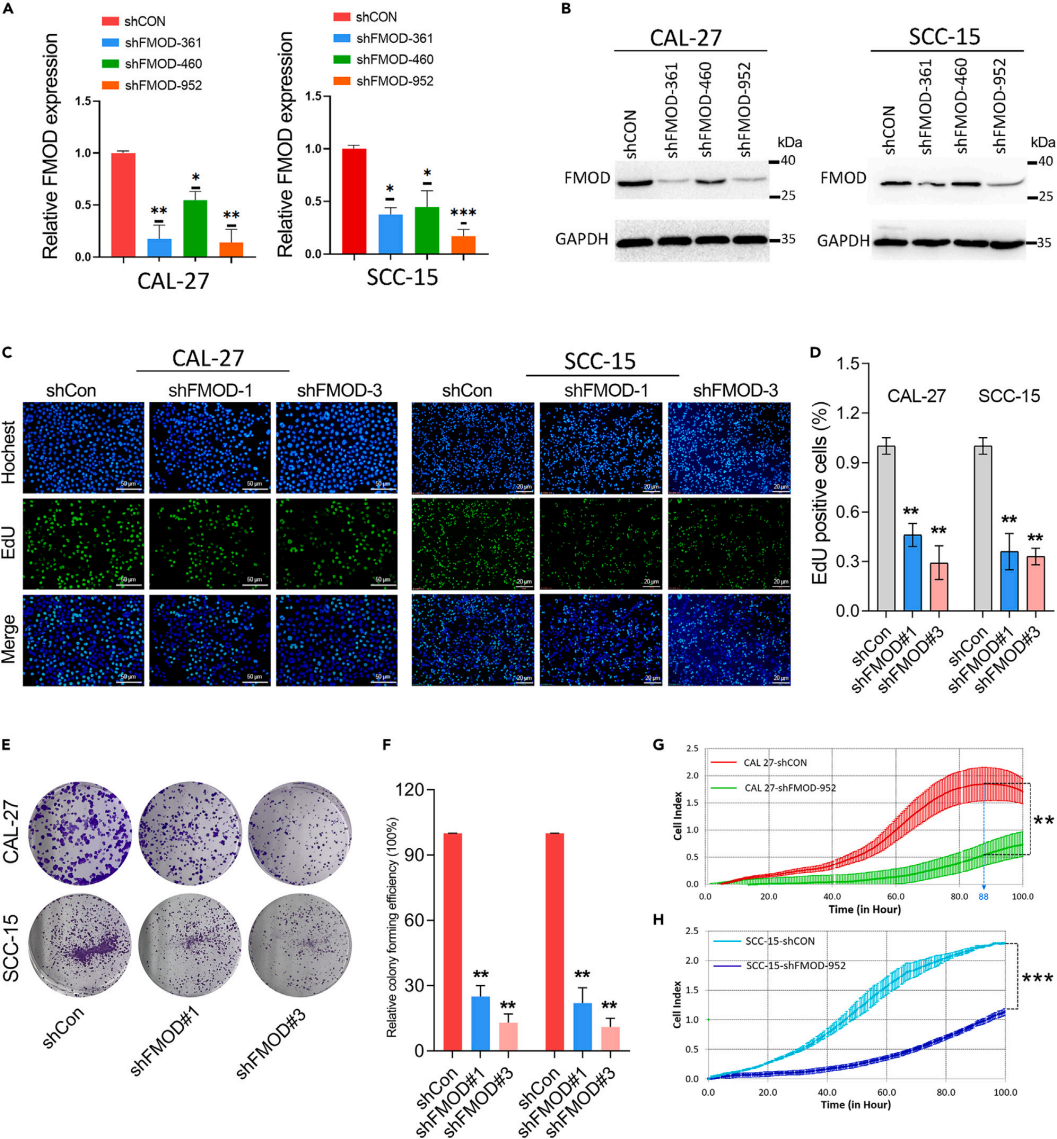
FMOD knockdown significantly inhibits OSCC cell proliferation (A and B) The knockdown efficiency of FMOD was determined in OSCC cell lines by qRT-PCR and western blotting assays. (C and D) EdU assay was performed to detect the effect of FMOD knockdown on OSCC cell proliferation. The nuclei of all cells were labeled with Hoechst dye, and proliferative nuclei were labeled with Azide 488 dye. (E and F) A colony formation assay was performed to examine the effect of FMOD knockdown on OSCC cell colony formation. (G and H) RTCA assay was conducted to observe the effect of FMOD knockdown on OSCC cell growth. OD values of cells in each group were detected every 30min for 100h.。 ∗, p < 0.05; ∗∗, p < 0.01; ∗∗∗p < 0.001.

Based on the clinical analysis, FMOD was overexpressed and may function oncogenic roles in OSCC. Therefore, loss-of-function studies were conducted to validate the biological role of FMOD in OSCC. To avoid the target-off effect, we designed and constructed three shRNA lentiviruses targeting different regions of the FMOD transcript for knockdown of FMOD expression. According to the results of qRT-PCR and western blotting assays, the stably transfected cell lines using shRNA#1 and shRNA#3 lentivirus were selected for subsequent experiments (Figures 2A and 2B).

To observe the effect of FMOD-depletion on the growth and proliferation of OSCC cells, EdU assay, colony formation assay, and Real Time Cellular Analysis (RTCA) were performed in OSCC cell lines, respectively. The EdU assay showed that the replication activity of DNA in FMOD knockdown cells was significantly lower than that in the control group (Figures 2C and 2D). Similarly, the colony formation assay verified that the knockdown of FMOD obviously decreased the colony formation capabilities of OSCC cells (Figures 2E and 2F). Furthermore, RTCA results showed that FMOD depletion significantly decreased the proliferation rates of OSCC cell lines (Figures 2G and 2H). Taken together, FMOD is required for cell proliferation of OSCC cells.

### Knockdown of FMOD inhibits OSCC migration and invasion *in vitro* and *in vivo*

**Figure 3:**
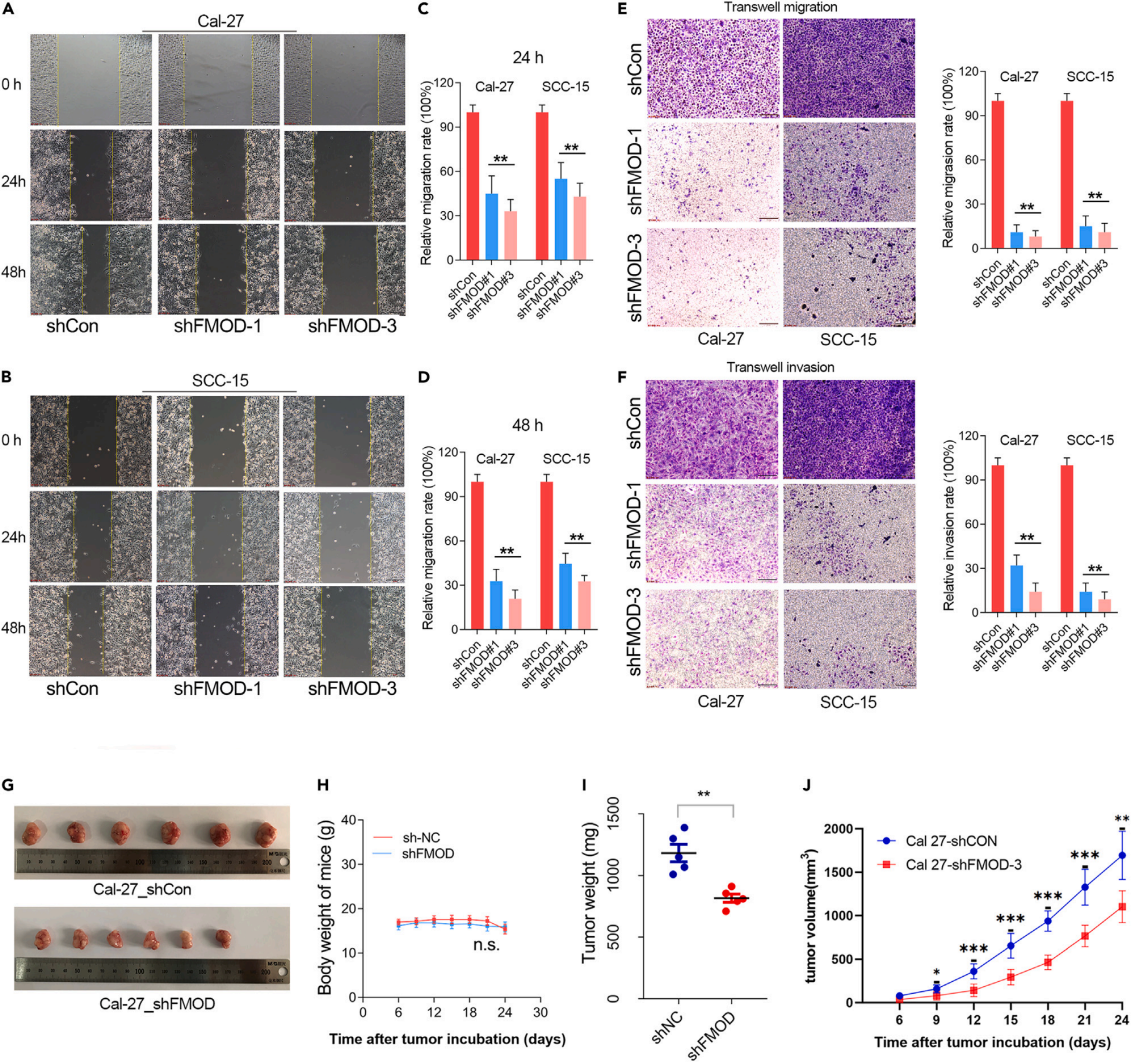
Knockdown of FMOD significantly hindered OSCC progression *in vivo* and *in vitro* (A and B) Scratch wound healing assays showed that knockdown of FMOD inhibited OSCC cell migration. (C and D) Transwell migration assay showed that knockdown of FMOD inhibited OSCC cell migration. (E and F) Transwell invasion assay showed that knockdown of FMOD inhibited OSCC cell invasion. (G) The morphology of nude mice and transplanted tumors. Subcutaneous xenograft models were performed in nude mice using OSCC cells. (H) The weight of nude mice in each group at different time points. (I) The weight of xenograft tumor in each group after euthanasia in nude mice. (J) The volume of xenograft tumors in each group at different time points. ∗, p < 0.05; ∗∗, p < 0.01; ∗∗∗p < 0.001.

Given that FMOD expression showed a significant association with lymph node metastasis in OSCC, we further investigated the role of FMOD depletion in OSCC cell metastasis using wound healing and transwell Matrigel assays. The wound healing assay showed that the number of migrating cells in the FMOD knockdown group was significantly lower than that in the control group at both 24 and 48 h time points (Figures 3A and 3B), suggesting FMOD knockdown inhibited OSCC cell migration. Consistently, the transwell migration assays revealed that the knockdown of FMOD significantly inhibited cell migration in OSCC cell lines (Figures 3C and 3D). In addition, the transwell invasion assays indicated that FMOD depletion significantly repressed the invasion abilities of OSCC cell lines (Figures 3E and 3F). These findings together indicated that FMOD acts as an oncogene in OSCC progression and metastasis *in vitro*.

To verify the oncogenic role of FMOD in driving OSCC progression *in vivo*, we established a xenograft OSCC tumor model by subcutaneous injection of CAL27-shFMOD-3 and CAL-27-shCON cells in nude mice and examined the growth of tumors. The results showed that FMOD knockdown resulted in significant decreases in the tumor weight and volumes (Figures 3G–3J). Compared to the control group, the growth rate of transplanted tumors in the FMOD knockdown group was significantly inhibited, with a decrease of up to 50% from day 6 to day 18 and a decrease of 30%–40% from day 18 to day 24 (Figure 3J). These results suggested that FMOD promotes OSCC progression *in vivo* and *in vitro*.

### Depletion of FMOD leads to inhibition of EGFR signaling in OSCC

**Figure 4:**
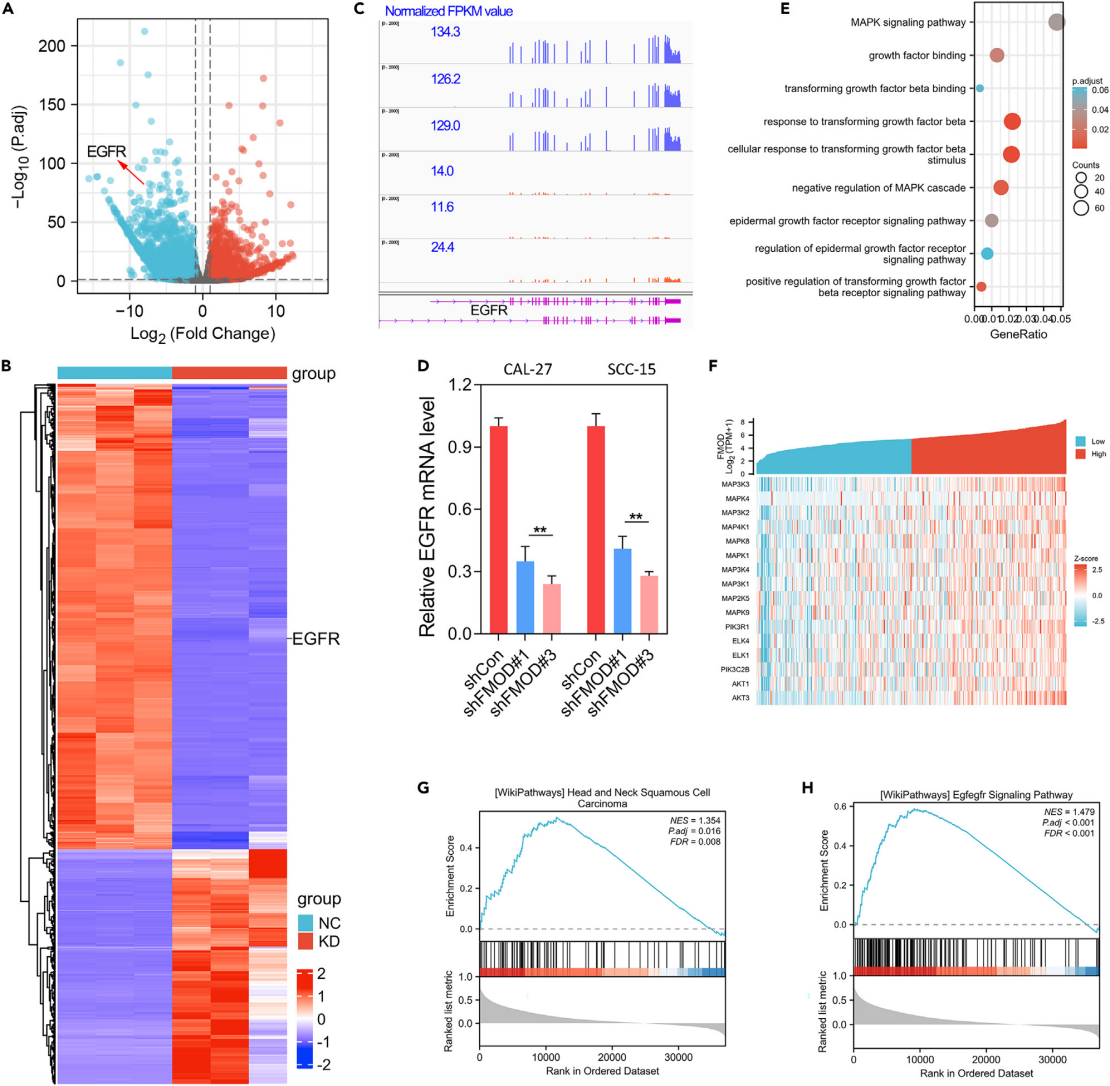
FMOD knockdown showed a profound effect on EGFR expression and signaling (A) The differentially expressed genes (DEGs) after FMOD depletion were shown in the volcano plot. (B) The heatmap reveals the DEGs (log2FC > 2, p < 0.05) after FMOD knockdown. (C) RNA-seq analysis revealed the transcripts’ abundance of EGFR after FMOD knockdown. (D) The RNA-seq data was verified using qRT-PCR assay. The EGFR mRNA level was significantly decreased after FMOD knockdown. (E) GO/KEGG analysis based on the RNA-seq data (GSE227643) showed that DEGs by FMOD knockdown were enriched in the EGFR and the MAPK signaling axis. (F) Gene expression correlation analysis was performed. FMOD was highly co-expressed with MAPK signaling pathway. (G and H) GSEA analysis based on the gene highly co-expressed with FMOD showed that FMOD was involved in HNSCC and EGFR signaling. ∗∗, p < 0.01.

To uncover the underlying molecular mechanisms of FMOD in driving OSCC progression, RNA sequencing studies (GSE227643) were performed in the FMOD-silenced OSCC cells. The differentially expressed genes (DEGs) were analyzed using DEseq2 R package. The significant DEGs were shown in the volcano plot and heatmap (Figures 4A and 4B). Epidermal growth factor receptor (EGFR), which functioned as an oncogene in multiple cancers, was significantly downregulated after FMOD knockdown (Figures 4A–4C). To validate the RNA-seq data, qRT-PCR assays were further conducted in FMOD-inhibited OSCC cell lines. The results showed that EGFR mRNA level was significantly decreased in FMOD knockdown cell lines (Figure 4D). GO/KEGG analysis based on the RNA-seq data of FMOD knockdown showed that the DEGs were enriched in EGFR and TGF-beta signaling (Figure 4E).

On the other hand, gene expression correlation analysis was performed based on the gene expression data in TCGA_OSCC cohort. The genes highly co-expressed (R > 0.2, p < 0.001) with FMOD were further collected to conduct GSEA analysis. The gene expression analysis showed that FMOD was highly co-expressed with MAPK signaling-related genes (Figure 4F). In addition, GSEA analysis showed that the genes highly co-expressed with FMOD were enriched in HNSCC and EGFR signaling pathways (Figures 4G and 4H). These results together suggested that FMOD may promote HNSCC through EGFR signaling pathway.

**Figure 5:**
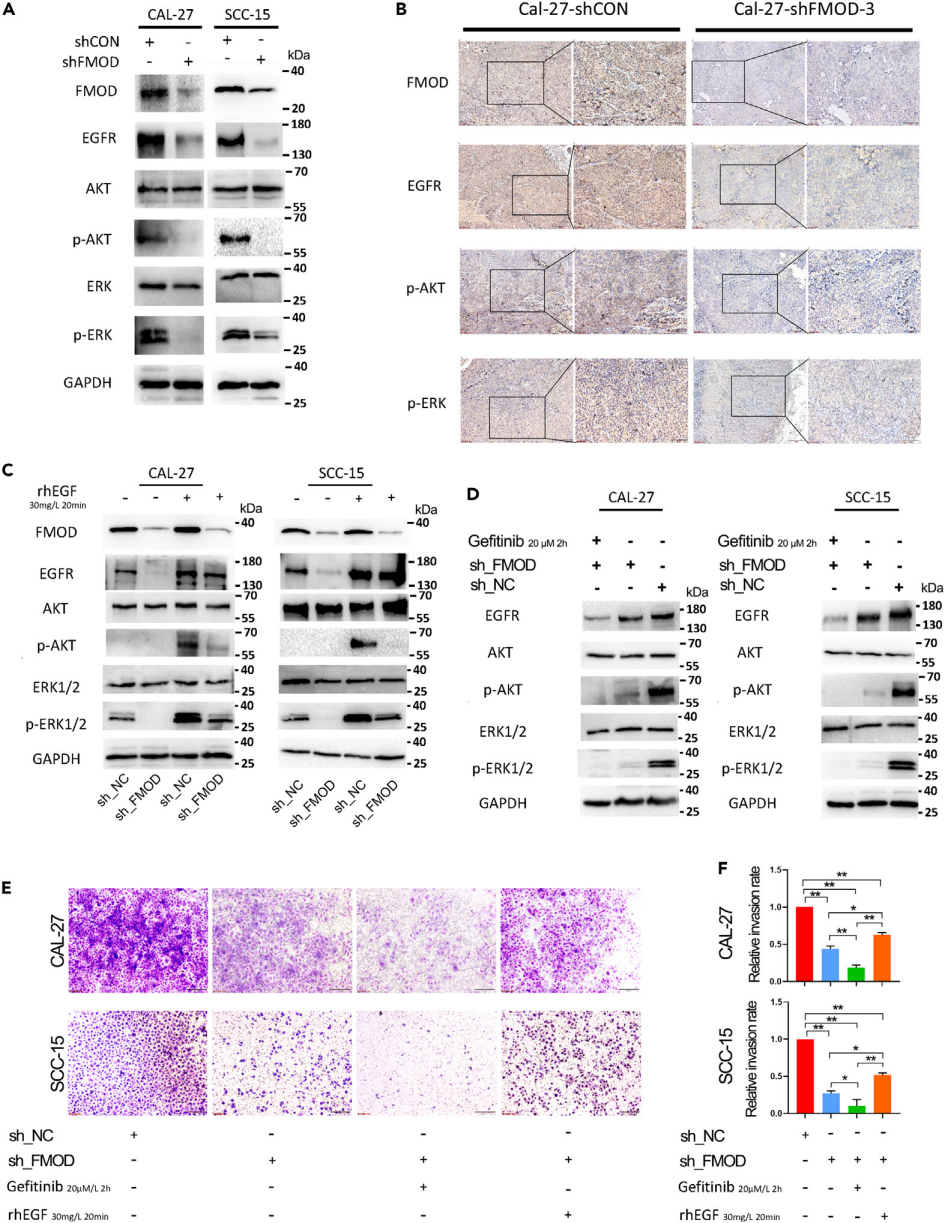
FMOD promotes OSCC progression through the activation of EGFR signaling (A) WB analysis of EGFR and downstream AKT and MAPK signaling after FMOD knockdown in OSCC cell lines. (B) IHC assay using xenograft tumor showed that FMOD knockdown inhibited EGFR and downstream AKT and MAPK signaling *in vivo*. (C) Rescue WB assay confirmed that exogenous recombinant EGF protein partially restored the inhibitory effect of FMOD knockdown on the EGFR signaling pathway. (D) Gefitinib aggravated the inhibitory effect of FMOD knockdown on the EGFR signaling pathway. (E and F) Rescue transwell assay confirmed that FMOD promotes OSCC progression via the EGFR signaling pathway. ∗, p < 0.05; ∗∗, p < 0.01; ∗∗∗p < 0.001.

To confirm the proposed probability, we performed western blotting in OSCC cell lines to detect the effect of FMOD knockdown on EGFR-related signaling pathways. The results showed that FMOD depletion resulted in decreased protein levels of EGFR and corresponding downstream phosphorylated AKT and ERK (Figure 5A). In other words, FMOD depletion inhibited EGFR/AKT or EGFR/ERK axis in OSCC. Moreover, IHC assay using xenograft OSCC tumor showed that FMOD knockdown inhibited OSCC progression through suppressing EGFR/AKT or EGFR/ERK signaling axis (Figure 5B). Rescue assays showed that the inhibitory effect of FMOD knockdown on the downstream signal pathway of EGFR can be partially restored by the exogenous EGF recombinant protein, and further enhanced by the EGFR inhibitor Gefitinib (Figures 5C and 5D). Rescue transwell invasion assays further confirmed that FMOD promotes OSCC progression dependent on activated EGFR/AKT or EGFR/ERK axis (Figures 5E and 5F).

### FMOD is directly targeted by miR-338-3p in OSCC

**Figure 6:**
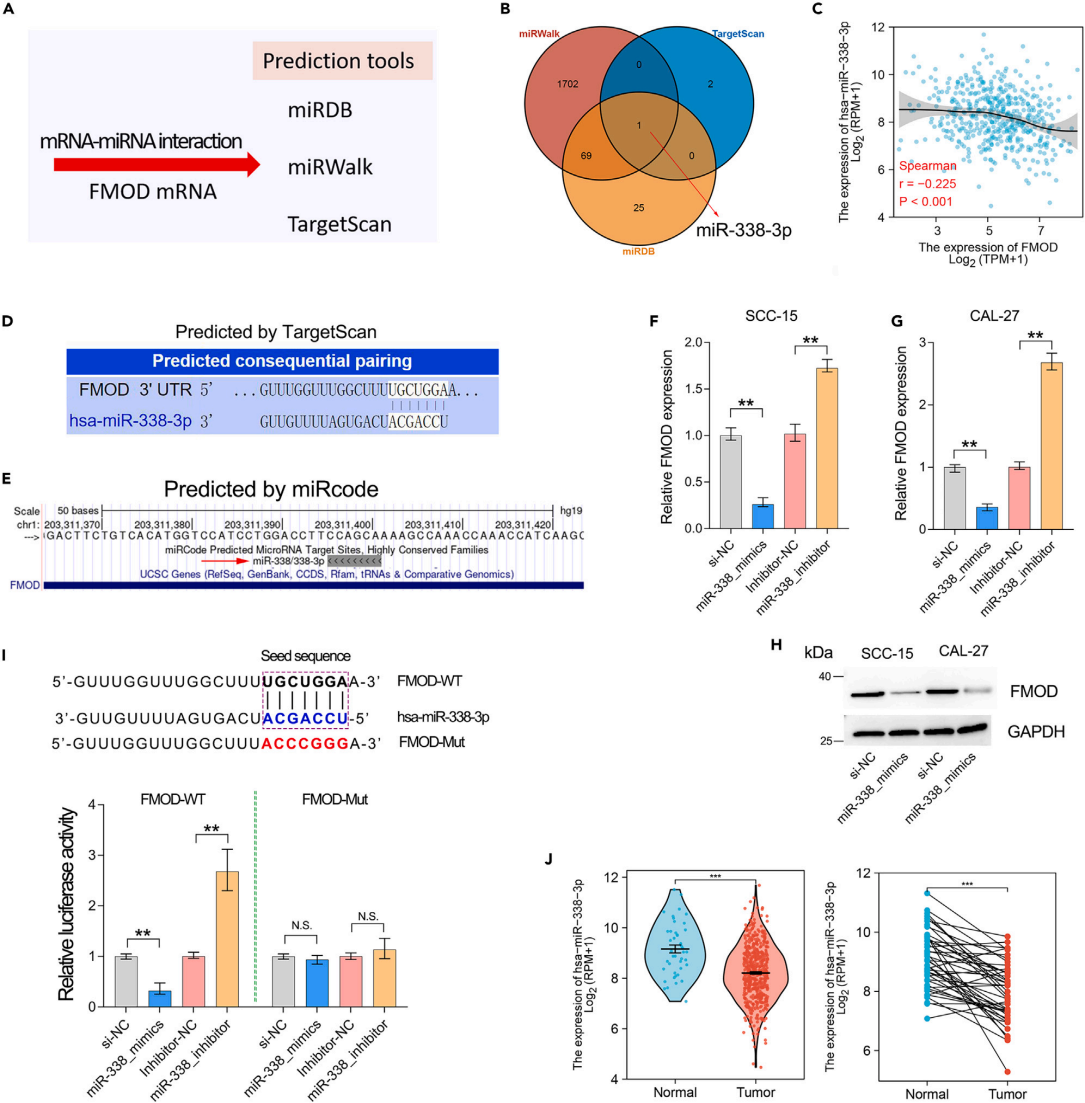
FMOD is directly targeted by has-miR-338-3p in OSCC (A) Multiple prediction tools were performed to explore the potential miRNAs targeting FMOD. (B) Has-miR-338-3p was the only miRNA predicted to interact with FMOD. (C) FMOD expression was negatively correlated with FMOD expression. (D and E) The binding site in FMOD transcript sequence was shown in different prediction tools. (F and G) FMOD mRNA expression was downregulated by miR-338-3p mimics and upregulated by miR-338-3p inhibitors in OSCC cell lines. (H) FMOD protein level was decreased by miR-338-3p mimics in OSCC cell lines. (I) Luciferase assay showed that the binding site of miR-338-3p in FMOD transcript sequence is required for the negative regulation of FMOD by miR-338-3p. (J) miR-338-3p was significantly downregulated in OSCC. ∗∗, p < 0.01; ∗∗∗, p < 0.001.

Given the elevated levels of FMOD mRNA and protein in OSCC, we further explored the reasons for the overexpression of FMOD. It is well known that microRNA generally negatively regulates gene expression at the transcriptional level. Therefore, we explored the potential miRNAs that may be involved in regulating FMOD mRNA levels. miRNA negatively regulates gene expression depending on the binding of its seed sequence to the target gene transcript. For this reason, we used various prediction tools to predict the binding sites of miRNA seed sequences contained in FMOD transcripts (Figure 6A). miR-338-3p was the only miRNA predicted by all tools to target FMOD (Figure 6B). Coincidentally, gene expression correlation analysis showed that miR-338-3p was significantly negatively correlated with FMOD mRNA level in OSCC cohort (Figure 6C). Furthermore, FMOD transcript sequence contains the binding site of the miR-338-3p seed sequence (Figures 6D and 6E). Taken together, FMOD might be a putative target gene of miR-338-3p in human cells.

To confirm this possibility, we transfected miR-338-3p inhibitors and mimics in OSCC cell lines. The qRT-PCR assays further verified that FMOD mRNA expression was increased by miR-338-3p inhibitor but decreased by miR-338-3p mimics in OSCC cell lines, suggesting that FMOD was negatively regulated by miR-338-3p in OSCC (Figures 6F–6H). Additionally, the luciferase reporter assay showed that FMOD was directly targeted by miR-338-3p since the inhibitory effect of miR-338-3p on FMOD expression was dependent on the interaction of FMOD and miR-338-3p (Figure 6I). In order to further determine whether overexpression of FMOD transcripts is caused by miR-338, we further analyzed the expression of miR-338 in OSCC cancer and adjacent tissues. The results showed that miR-338-3p was significantly downregulated in OSCC (Figure 6J).

Taken together, we herein the mapped the working model of pro-tumor role of FMOD in OSCC. In OSCC, miR-338-3p was significantly downregulated. The decreased miR-338 weakens its negative regulation of FMOD expression, resulting in FMOD overexpression. Finally, FMOD overexpression promotes OSCC progression by activating the oncogenic EGFR signaling pathways. Our research work highlights that targeting FMOD may improve the prognosis of OSCC patients via inhibiting EGFR signaling pathways.

## Discussion

As an ECM protein, Fibromodulin (FMOD) plays a role in multiple cellular biological events, including ECM remodeling, collagen fibrogenesis, dedifferentiation, and inflammation.^19^^,^^20^ Beyond that, current evidence suggests that FMOD acts as diagnostic and prognostic biomarkers in cancers and exerts oncogenic roles by regulating cell apoptosis, angiogenesis, and migration.^20^^,^^21^ Mayr and colleagues have reported that FMOD is a tumor-associated antigen in chronic lymphocytic leukemia via facilitating the expansion of specific CD8^+^ autologous T lymphocytes.^22^ Ao Zhi et al. found that FMOD expression was positively correlated with tumor angiogenesis in small-cell lung cancer.^12^ Recently, Sengupta et al. found that FMOD promotes glioma angiogenesis and growth by activating integrin-dependent Notch signaling in endothelial cells.^23^ Correspondingly, targeting cell surface protein FMOD has been found to be a promising therapeutic approach for triple-negative breast cancer, colorectal cancer melanoma, and chronic lymphocytic leukemia.^24^^,^^25^^,^^26^^,^^27^^,^^28^ Nevertheless, the expression signature, clinical significance, and biological role of FMOD in OSCC remain largely unclear.

In this study, we identified that FMOD expression is overexpressed in OSCC and showed a significant association with clinical tumor stage (p = 0.011) and lymph node metastasis (p = 0.032). Although the OSCC samples included in this study do not contain prognostic follow-up information, studies by Pourhanifeh et al. have confirmed that FMOD overexpression is significantly associated with poor overall survival prognosis in HNSCC.^29^ That means FMOD can be used as a diagnostic and prognostic marker for OSCC. Notably, our finding highlights that FMOD was a direct target gene of miR-338-3p, which functioned as a tumor repressor in cancers, especially OSCC.^30^^,^^31^ Therefore, it is reasonably speculated that the FMOD overexpression may be caused by the downregulation of miR-338-3p in OSCC.

On the other hand, we further explored the biological role of FMOD in OSCC *in vivo* and *in vitro*. Consistent with clinical analysis results, FMOD depletion resulted in an obvious inhibitory effect of cell proliferation and metastasis in OSCC cell lines. More importantly, we confirmed the pro-tumor effects of FMOD *in vivo* using OSCC cells (CAL-27) xenograft in nude mice. Based on the clinical analysis and *in vivo* and *in vitro* studies, there is no doubt that FMOD acts as an oncogene in OSCC.

To further uncover the molecular mechanism of FMOD in OSCC, we performed high-throughput transcriptome sequencing studies in FMOD-silenced OSCC cells. The results showed that the EGFR expression level was significantly reduced by FMOD knockdown. Likewise, GO/KEGG analysis based on the RNA-sea data of FMOD knockdown and GSEA analysis based on the TCGA_OSCC together indicated that FMOD depletion showed a significant effect on EGFR signaling axis. Besides, WB and IHC assays also confirmed that EGFR, *p*-Akt, *p*-ERK expression was inhibited by FMOD knockdown. Moreover, rescue assays further confirmed that FMOD promoted OSCC progression through EGFR signaling pathway (Figures 5C–5F).

Cancer cells can aberrantly express and secrete cytokines and growth factors, such as epidermal growth factor (EGF), VEGF, and fibroblast growth factor (FGF) that act in an autocrine, paracrine, or juxtacrine manner to affect tumor environment and facilitate tumor progression.^32^ As a member of the ErbB family of receptors, epidermal growth factor receptor (EGFR) activated downstream intracellular tyrosine kinase signaling via binding to endogenous ligands such as EGF or transforming growth factor α.^33^ Increasing studies have reported that EGFR and its endogenous ligands were overexpressed and exert pro-tumor roles in HNSCC.^34^^,^^35^ EGFR overexpression is clinically associated with poor prognosis and resistance to radiation therapy in HNSCC.^36^ As a result of this, blockade EGFR signaling became a promising strategy to overcome HNSCC growth and radiology resistance.^37^^,^^38^ In this work, we uncovered an essential role of FMOD in the activation of the EGFR signaling axis. Targeting FMOD may be an effective strategy for inhibiting the EGFR pathway in OSCC patients.

In conclusion, FMOD overexpression is clinically associated with malignant progression and lymph node metastasis in OSCC patients. Our finding highlights that FMOD overexpression drives OSCC progression through the activation of the EGFR signaling axis. In addition, we uncover a possible mechanism for FMOD overexpression: Hsa-miR-338-3p downregulation weakens its negative regulation of the target gene FMOD, resulting in its overexpression.

### Limitations of the study

Our findings implicated that FMOD depletion plays a profound role on EGFR signaling, but the way in which FMOD affects EGFR transcript levels is unclear. As we observed that FMOD protein is mainly localized in the cytoplasm of the SCC15 cell line, there is a possibility that FMOD can serve as a transcription factor. Nevertheless, there is also a possibility that FMOD indirectly regulates EGFR transcription through other pathways. Further exploration is needed on how FMOD regulates EGFR mRNA and protein expression. In addition, further experiments are needed to clarify the differences in subcellular localization of FMOD between two OSCC cell lines.

## STAR★Methods

### Key resources table

**Table:** 

REAGENT or RESOURCE	SOURCE	IDENTIFIER
**Antibodies**		

anti-FMOD, mouse polyclonal	Bioss	Cat #bs-12362R
anti-EGFR,rabbit monoclonal	Proteintech	Cat #66455-1-Ig
anti-Phospho-AKT (Ser473),rabbit monoclonal	Proteintech	Cat #66444-1-Ig
anti-Phospho-ERK1/2 (Thr202/Tyr204),rabbit monoclonal	Proteintech	Cat #28733-1-AP
anti-AKT, rabbit polyclonal	Proteintech	Cat #10176-2-AP
anti-ERK1/2, rabbit polyclonal	Proteintech	Cat #11257-1-AP
anti-FMOD, rabbit polyclona	Proteintech	Cat #13281-1-AP
anti-GAPDH,mouse monoclonal	Proteintech	Cat #60004-1-lg
goat anti-rabbit	Biosharp	Cat #BL003A
Alexa Fluor 488 donkey anti-mouse IgG	Proteintech	Cat #SA00014-3

**Biological samples**		

Patients with OSCC oral tissue	Taihe hospital	N/A
Patients with mandibular third molars Normal oral gingival tissue	Taihe hospital	N/A

**Chemicals, peptides, and recombinant proteins**		

DMSO	Solarbio	Cat #D8371
Ham‘s F12 nutrient medium (DMEM/F12)	Pricella	Cat #PM150312
fetal bovine serum (FBS)	Gibco	Cat # 16140071
RIPA buffer	Applygen	Cat #C1053-100
Enhanced Chemiluminescence	Epizyme	Cat #G415DA0001

**Critical commercial assays**		

PrimeScript*™* RT reagent kit	Takara	Cat #RR037A
TB Green*®* Premix Ex Taq*™* kit	Takara	Cat # RR420A
TRizon-RNA Extract kit	CWBIO	Cat # CW0580
Bicinchoninic acid kit	Biosharp	Cat #BL521A

**Deposited data**		

GEO database	GSE227643	N/A

**Experimental models: Cell lines**		

Human:CAL-27 cells	Shanghai Hongshun TeChinaology	N/A
Human :SCC-15 cells	Shanghai Hongshun TeChinaology	N/A
Human :HOK-8 cells	Affiliated Stomatology Hospital of Guangzhou Medical University	N/A

**Experimental models: Organisms/strains**		

Mouse: BALB/c-nude	Animal Center of Hubei University of Medicine	N/A

**Oligonucleotides**		

siRNA targeting sequence: shFMOD-Homo-NC: TTCTCCGAACGTGTCACGT	This paper	N/A
siRNA targeting sequence: shFMOD-Homo-361: GGCCATGTACTGTGACAATCG	This paper	N/A
siRNA targeting sequence: shFMOD-Homo-460: GGAAGGCGTCTTTGACAATGC	This paper	N/A
siRNA targeting sequence: shFMOD-Homo-952: GGCCTCCAACACCTTCAATTC	This paper	N/A
qPCR primers:GAPDH forward: 5'- AGGTCGGTGTGAACGGATTTG-3' Reverse: 5'-TGTAGACCATGTAGTTGAGGTCA-3'	This paper	N/A
qPCR primers:FMOD forward: 5'-TACCTCCGCAGCCAGCAGTC-3' Reverse: 5'GGATGGAGAGCCGTAGGTGTAGG-3'	This paper	N/A

**Software and algorithms**		

ImageJ	Schneider et al.^7^	https://imagej.nih.gov/ij/

### Resource availability

#### Lead contact

• Further information and requests for resources and reagents should be directed to and will be fulfilled by the lead contact, Weidong Leng (lwd35@163.com).

#### Materials availability

• This study did not generate new unique reagents.

### Experimental model and study participant details

#### Tissue samples

All tissue samples were collected from a total of 77 patients with OSCC who underwent surgical resection from January 10, 2017, to July 15, 2020, in Taihe Hospital. Normal oral gingival tissue samples were collected from 15 patients who underwent extraction of mandibular third molars during the same time period. Patients under 18 years old, with autoimmune diseases, previous history of malignant tumor, previous chemotherapy, radiotherapy, surgery, alternative drug therapy, immune deficiency, autoimmune diseases, hepatitis, human immunodeficiency virus infection, pregnancy, or breastfeeding were excluded from this study. All tissue samples were formalin-fixed, paraffin-embedded, and sectioned in the Department of Pathology, Taihe Hospital. Clinic-pathological information of the OSCC patients, including sex, age, smoking history, alcohol history, TNM stage, and histological grade, was also collected. Written informed consent forms were obtained from all patients. The research was approved by the Ethics Committee of Taihe Hospital, Hubei University of Medicine.

#### Cells

Human oral squamous cell carcinoma cell lines CAL-27 and SCC-15 and human normal oral keratinocytes HOK-8 were were cultured with DMEM (Procell, China) supplemented with 1% penicillin-streptomycin and 10% fetal bovine serum (Gibco, US) in an incubator (Thermo Fisher, USA) supplemented with 5% CO_2_ at 37°C.

#### Animals

The four-week-old female Balb/C-nude mice were reared in SPF environmental cages. The Xenograft tumorigenic experiment was performed as we previously described.^39^^,^^40^ The mice were randomly divided into 2 groups, with 6 mice in each group, CAL-27-shCON and CAL-27-shFMOD-952 cells (100ul, 1×10^6^ cells per/animal) were inoculated into the left back subcutaneously of mice. The tumor tissues were observable on the sixth day after inoculation. Then, the tumor size and weight of mice were measured every three days. On the 24th day after inoculation, the mice were sacrificed by neck dislocation. The tumor tissues were collected and recorded by photography. Tumor volumes were calculated according to the formula A × B2/2 (A= maximum diameter; B = minimum diameter) (mm^3^). The isolated grafts were fixed with 4% paraformaldehyde fixative for 24 hours, embedded into paraffins, and then made into paraffin sections for immunohistochemical staining.

### Method details

#### Immunohistochemistry staining and evaluation

Expression of target proteins in the 4μm sections from tissue samples of the patients and xenograft tumor tissues of BABL/ C-NU mice was determined using immunohistochemistry staining (IHC) according to the published procedure.^12^ Primary antibodies and dilutions for IHC included FMOD (Bioss, China) in 1:500 dilution, EGFR in 1:1000 dilution (Proteintech, China), p-AKT (Proteintech, China) 1:200, and p-Erk1/2 (Thr202/Tyr204) in 1:200 (Proteintech, China). Goat anti-rabbit or goat anti-rat antibodies conjugated with HRP were used as secondary antibodies. Expression levels of FMOD were quantified using the scores for staining intensity and the scores for the percentage of positive cells. Scores for staining intensity: 0 = no staining, 1 = weak staining, 2 = medium staining, 3 = strong staining. scores for the percentage of positive cells, score 0 = 0%∼ 5% positive cells, 1 = 6%∼ 25% positive cells, 2 = 26%∼ 50% positive cells, 3 = 51%∼ 75% positive cells and 4=76%∼100%. The expression levels of FMOD were quantified by the product of the scores for staining intensity and the scores for the percentage of positive cells: 0=negative, 1∼4=weak, 5∼8=medium, 9∼12=strong.

#### Lentiviral gene-knockdown constructs and transfection

The transfection assay was performed as previously described.^39^^,^^41^^,^^42^^,^^43^ Briefly, FMOD short hairpin RNA (shRNA) fragments were designed, synthesized, and inserted into LV10N (U6/mCherry&Puro) (GenePharma, Suzhou, China) to knock down the expression of FMOD in cells. CAL-27 and SCC-15 cells were infected with lentiviral constructs which were adjusted to 1 ×10^8^ TU/mL for both LV10N-shFMOD and LV10N-shCON. Stable infected cells were selected using puromycin (2 ug/mL).

#### Immunofluorescence assay

OSCC cells were seeded in 10-mm confocal dishes with a density 1 × 10^5^/well. Immunofluorescence assay was performed by following the published method.^44^ Goat anti-mouse FMOD (Proteintech, China) was used as the primary antibody, and Alexa Fluor 488 donkey anti-mouse IgG (Proteintech, China) was used as the secondary antibody. The results were observed using a laser scanning confocal microscope (Olymbus FV3000RS, Japan). The experiment was independently repeated three times.

#### Western blot analysis

OSCC cells were lysed using RIPA buffer (Applygen, China) on ice. The total protein of the lysates was quantified using BCA kit (Biosharp, China) reagents. Twenty micrograms of lysate of each sample were loaded and resolved in 10% SDS-PAGE gel by electrophoresis. The resolved proteins were transferred onto the PVDF membranes and blocked in TBST buffer containing 10% nonfat milk, then incubated with primary antibodies overnight. After washing with TBST buffer three times, the membranes were incubated with secondary antibodies for 3 h. After three washes with TBST buffer, the membranes were developed with Enhanced Chemiluminescence (ECL) (Epizyme, China). The signals were recorded using a gel imaging analysis (GE, USA). ImageJ software was used to measure the density of bands. The relative protein levels were calculated using GAPDH as the internal control. Primary antibodies were GAPDH (1:5000, 60004-1-lg), FMOD (1:1000, 13281-1-AP), EGFR (1:5000, 18986-1-1AP), AKT (1:2500, 10176-2-AP), p-Akt (1:1000, 28731-1-1AP), ERK1/2 (1:2000, 16443-1-1AP), and p-ERK1/2 (Thr202/Tyr204) (1:2000, 28733-1-1AP) were purchased from Proteintech, China. Second antibodies(1:10000，BL003A) was purchased from Biosharp, China.

#### Cell proliferation assay

The proliferation of OSCC cells was determined using real-time cell analysis (RTCA) and EdU assay. To be performed for the RTCA assay, the cells were seeded (5×103 cells per well) into E-plates and cultured at 37°C, and 5% CO_2_ for 100 h, and data were obtained using RTCA xCELLigene system (ACEA Biosciences Inc, USA). To perform EdU assay, cells (1×10^5^ cells per well) were seeded into 96-well plates, and cell proliferation was detected using BeyoClickTM EdU-488 proliferation detection kit (Beyotime BioteChinaology, China) by following the manufacturers’ protocol.

#### Cell migration assay

The cell migration ability was evaluated by scratch wound experiments. The cells (1×10^6^ cells per well) were seeded in 6-well plates. After the cells reached 90% confluence, a linear wound was drawn in the cell layer with the tip of a 200 μl pipette nozzle, after being cleaned twice with PBS, a serum-free culture medium was used for 48 hours. Images were taken at 0 h, 24 h, and 48 h under the inverted fluorescence microscope. Scratch width was measured, and cell mobility was calculated according to the formula (A - B) / A (A: scratch width at 0 h, scratch width at 24 h (48 h).

#### Cell invasion assay

Cell invasion was measured using a 24-well transwell plate (BD Biosciences, USA), 8-μm well, coated with Matrigel (1 mg/mL; BD Biosciences). The cells (3×10^5^ cells per well) were plated in the upper chamber of serum-free medium, and the 500 μl complete medium was added in the lower chamber, after 48 hours of culture. The upper chamber was cleaned with PBS 3 times, fixed with paraformaldehyde, and stained with crystal violet dye (Servicebio, China) for 30 minutes. After the upper chamber membrane cells were wiped with cotton swabs, the outer membrane cells were photographed.

OSCC cells stably infected with CAL27-shFMOD-952 and CAL-27-shCON cells were collected (three samples per cell type). RNA samples of these cells were extracted using the TRizon-RNA Extract kit（CWBIO, Chian）. The transcriptome profiles of these samples were detected using RNA sequencing analysis in Wuhan Bena Technology Co., LTD. After data normalization and clean-up, differentially expressed genes in all three FMOD-knockdown cell lines were identified using DESeq2 (version: 1.26.0). GO terms and KEGG pathways enrichment analysis were performed as we previously described.^39^^,^^45^ Differential genes generate heat maps using R software. The RNA-seq data of FMOD knockdown generated in this study are available in the GEO repository (GSE227643).

### Quantification and statistical analysis

The correlation between the expression level of FMOD and clinical parameters of OSCC patients was analyzed by the Pearson Chi-square test. The gray value of WB images was calculated by ImageJ software. Graphing analysis of data from cell and animal experiments was performed using GraphPad Software, and the significance between comparison groups was determined by two-tailed pairing, unpaired T-test, or conventional one-way ANOVA. Data in the figure are expressed as mean ± standard deviation (SD). A difference of P < 0.05 was considered statistically significant. All experiments were performed at least three times.

## Data Availability

• The RNA-seq data reported in this paper has been deposited at GEO and publicly available as of the date of publication. Accession numbers are listed in the key resources table. • This paper does not report original code. • Any additional information required to reanalyze the data reported in this paper is available from the lead contact upon request.
